# Promoting Effect of Soluble Polysaccharides Extracted from *Ulva* spp. on *Zea mays* L. Growth

**DOI:** 10.3390/molecules27041394

**Published:** 2022-02-18

**Authors:** Ragaa A. Hamouda, Mervat H. Hussein, Noura El-Ahmady El-Naggar, Mohammed A. Karim-Eldeen, Khalid H. Alamer, Muneera A. Saleh, Luluah M. Al Masoudi, Eman M. Sharaf, Reham M. Abd El-Azeem

**Affiliations:** 1 Department of Biology, College of Sciences and Arts Khulais, University of Jeddah, Jeddah 21959, Saudi Arabia; 2 Department of Microbial Biotechnology, Genetic Engineering and Biotechnology Research Institute, University of Sadat City, Sadat City 32897, Egypt; 3 Department of Botany, Faculty of Science, Mansoura University, Mansoura 35516, Egypt; mervathussen56@yahoo.com (M.H.H.); mohamed-aly@yahoo.com (M.A.K.-E.); 4 Department of Bioprocess Development, Genetic Engineering and Biotechnology Research Institute, City of Scientific Research and Technological Applications (SRTA-City), New Borg El-Arab City 21934, Egypt; nouraalahmady@yahoo.com; 5 Biological Sciences Department, Faculty of Science and Arts, King Abdulaziz University, Rabigh 21911, Saudi Arabia; kalamer@kau.edu.sa; 6 Department of Biology, Faculty of Sciences, Taif University, P.O. Box 11099, Taif 21944, Saudi Arabia; m.abdoo@tu.edu.sa (M.A.S.); lm.al-masoudi@tu.edu.sa (L.M.A.M.); 7 Bacteriology, Immunology and Mycology Dep., Animal Health Research Institute, Shebin El Kom 32511, Egypt; dr_emansharaf@yahoo.com; 8 Environmental Biotechnology Department, Genetic Engineering and Biotechnology Research Institute, University of Sadat City, Sadat City 32897, Egypt; reeham.abdelazeem@gebri.usc.edu.eg

**Keywords:** *Ulva fasciata*, *Ulva lactuca*, polysaccharides, protein, antioxidants, SDS PAGE, *Zea mays*

## Abstract

Seaweeds can play a vital role in plant growth promotion. Two concentrations (5 and 10 mg/mL) of soluble polysaccharides extracted from the green macroalgae *Ulva fasciata* and *Ulva lactuca* were tested on *Zea mays* L. The carbohydrate and protein contents, and antioxidant activities (phenols, ascorbic, peroxidase, and catalase) were measured, as well as the protein banding patterns. The soluble polysaccharides at 5 mg/mL had the greatest effect on the base of all of the parameters. The highest effects of soluble polysaccharides on the *Zea mays* were 38.453, 96.76, 4, 835, 1.658, 7.462, and 38615.19, mg/mL for carbohydrates, proteins, phenol, µg ascorbic/mL, mg peroxidase/g dry tissue, and units/g tissue of catalase, respectively. The total number of protein bands (as determined by SDS PAGE) was not changed, but the density of the bands was correlated to the treatments. The highest band density and promoting effect were correlated to 5 mg/mL soluble polysaccharide treatments extracted from *Ulva fasciata* in *Zea mays*, which can be used as a biofertilizer.

## 1. Introduction

The third most vital cereal crop in the world is maize (*Zea mays* L.), which comes after wheat, followed by rice [1]. Every portion of the maize plant has an efficient value, such as the grain, which is consumed in the production cooking oil; and the leaves, stalks, tassels, and cob, all of which can be consumed in animal feed and also used in the production of bioethanol [2,3]. The excess use of chemical synthetic fertilizers has led to the contamination of the soil and water basins, destroying micro-organisms and destroying friendly insects, which renders the crop more susceptible to infections and reduces soil fertility [4]. Biofertilizers are of the highest significance as a complement to synthetic chemical fertilizers in enhancing the nutrient materials provided to cereal crops due to the great nutrient turn-over in the cereal-yields system [5]. Algae could be a possible beneficial biomass to explore, as they are abundant and cheap [6,7]. The marine green alga *Ulva* sp. contains superior amounts of high-quality protein, carbohydrates, vitamins, and minerals [8,9]. Under salt stress, *Ulva lactuca* (ULF) enhanced the growth parameters of *Abelmoschus esculentus* due to the presence of nutrients and another important physiochemical compound besides growth hormones [10]. *Ulva lactuca* liquid fertilizers and combinations with various concentrations of zeolite were enhanced for the cucumber (*Cucumis sativus*) [11]. Safinaz and Ragaa [12] investigated whether the use of seaweeds as a bio-fertilizer improved the vegetative characteristics of corn plants. Dhargalkar and Untawale [13] deliberated the impacts of marine algal extracts on the growth of turnips and chillies, and showed that minor concentrations of seaweed liquid fertilizer (SLF) increased the seed germination rate. The fertilizer (SLF) contained macronutrients, trace metals, organic constituents such as amino acids, and plant growth regulators (PGR) like auxin, cytokinin, and gibberellins. Wajahatullah et al. [14] reported that algae and algae-derived products are extensively exhausted as additives in crop-yield systems due to the presence of several plant growth-promoting (PGP) compounds. Early seed germination, developed crop production, and enhanced resistance to both biotic and abiotic stress were all found to be beneficial effects of seaweed extract applications on plants [15]. Efficient elicitors of protection against plant disease are the results of the effects of polysaccharides extracted from algal extracts [16]. The growth and activities of microorganisms that were antagonistic to *Pythium ultimum*, a severe fungal pathogen that causes seedling damping-off disease, were estimated after liquid seaweed extracts were applied to the soil [17]. Polysaccharides are found in abundance in seaweeds [18,19]. When marine algae extracts were added to cabbage-cultivated soil, growth was estimated and microorganisms antagonistic to *Pythium ultimum* (serious pathogenic fungi) were enhanced [17]. Marine algae contain many types of polysaccharides [18,19]. Polysaccharides can act as prebiotic materials that enhance the growth of useful bacteria in the digestive tract, and provide growth enhancement and health-improving effects [20]. Ganapathy Selvam and Sivakuma [21] reported that the seaweed extracts of *U. reticulata* increase the crop productivity of *Vigna mungo* plants. Seaweeds liquid fertilizers are an effective eco-friendly approach for sustainable agriculture and the environment.

The aim of this study is to detect the effect of soluble polysaccharides extracted from *Ulva fasciata* and *Ulva lactuca* on growth parameters, carbohydrates, protein bands, and antioxidants such as the ascorbic acid, total phenolic, catalase, and peroxidase content of *Zea mays.*

## 2. Material and Methods

### 2.1. Algae

*U. facciata* and *U. lactuca* were gathered from the shallow water on the beach of the Mediterranean Sea at Abo Quire coast, Alexandria. The collected algae were cleaned using tap water and dried in an electric oven at 60 °C until they reached a constant weight.

### 2.2. Extraction of Water-Soluble Polysaccharides

The sulphated polysaccharide was extracted by the procedure detailed by Bourgougnon et al. [22]. Approximately 1500 mL of distilled water was added to 5 g each *U. fasciata* and *U. lactuca,* which was then heated to 80 °C for 4 h with magnetic stirring; after that, the suspension was cooled to 5 °C in a refrigerator, and filtered using Whatman no. 1 filter paper. Two volumes of absolute ethanol were added to the filtrate, with stirring. The precipitate was recovered, cleaned with ethanol, dehydrated with diethyl ether, and dried overnight at 50 °C.

### 2.3. Physical Characterizations

#### Thermal Characteristics of the Extracted Polysaccharides

The thermal stability of the extracted polysaccharide was investigated using thermogravimetric analysis (TGA) with a Shimadzu D-50 thermo-analyzer (Japan). The polysaccharide sample was heated at a rate of 15 °C/min in a dry nitrogen environment, and the thermal interference material was sintered alumina (α-alumina). The temperature was then measured with the aid of a Pt/Rh (10%) thermocouple.

The thermal stability of the extracted polysaccharide was investigated using thermogravimetric analysis (TGA) with a Shimadzu D-50 thermo-analyzer (Japan).

### 2.4. Chemical Characterizations

#### High-Performance Liquid Chromatography (HPLC)-Refractive Index Detector

The monosaccharide analysis of the extracts of the decomposed algae was performed by HPLC (Agilent 1100 HPLC system) using a Hypersil ASP-2 column (4.6 × 250 mm) with a mobile phase of acetonitrile–water (80:20) and a flow rate of 0.4 mL min^−1^. The column and optical unit were both heated to 35 and 40 °C, respectively. The injection volume of each integrated monosaccharide standard and the sample hydrolyses was 10µL.

### 2.5. Biological Activity

Investigation of the Promoting Growth Efficiency of the Extracted Water-Soluble Polysaccharides

#### 2.5.1. Plant Growth Analysis

The Ministry of Agriculture, Field Crop Institute, Agriculture Research Center, Giza, Egypt, provided seeds of *Zea mays* single cross 10. They were tested for size and shape uniformity before being surface-sterilized in 0.01 percent HgCl_2_ for 3 min [23]. They were then cleaned carefully with running distilled water. Following that, the seeds were soaked in soluble polysaccharides (10 mg mL^–1^, 5 mg mL^–1^) or water (control) for 4 h at 25 °C. The seeds were rinsed with sterile water for 15 min.

#### 2.5.2. Determination of Germination

The germination experiment was conducted as follows: The same number of seeds (10 seeds) were germinated in Petri dishes (11 cm) lined with two moist sheets of filter paper (Whatman no.1) and incubated in the dark at 25 °C for 5 days in order to determine the germination criteria, such as the percentage of seed germination, the radical length (the samples were ten replicates), and the amylase or protease activity (in triplicate). Another series of soaked seeds was grown in plastic pots (1 Kg sand soil capacity) and incubated under continuous illumination (72 µmol photon m^−2^ s^−1^) at 25 °C for 14 days; the sampling was performed on the seventh day and at the end of the experimental period (14 days). The growth criteria of the seedlings—such as shoot height and root length, leaf area, fresh and dry weight, and leaf area (ten replicates for each), as well as the photosynthetic pigment, carbohydrate, and protein content (triplicate sampling)—were determined. In addition, the antioxidant activities (triplicate sampling) were also determined.

### 2.6. Phytochemical Contents

The total carbohydrate contents were assessed with the phenol–sulphuric acid method, using glucose as the standard [24]. The protein content was investigated according to the method of Lowry et al. [25], using crystalline bovine serum albumin as the standard. The total phenol content was assessed by the Folin–Ciocalteu method [26].

#### 2.6.1. Total Soluble Proteins Banding Pattern for the Tested Crop Plant

The discontinuous polyacrylamide gel electrophoresis (SDS-PAGE) was exhausted for the protein analysis, allowing the method adopted by Stegman et al. [27] and Hemeida [28].

#### 2.6.2. Antioxidants

##### Total Ascorbic Acid Content

The total ascorbic acid content was assessed as indicated by Chen and Asada [29].

##### Catalase Activity

The CAT activity was assayed following the method of Kang and Saltveit [30].

##### Peroxidase

The activity was assayed following the method of Racusen and Foote [31].

##### Amylase Assay

The activity was assayed following the method of Monroe and Preiss [32].

##### Protease Assay

The protease was assayed according to Anson [33].

### 2.7. Statistical Analysis

The statistical analysis was conveyed by using ANOVA, analysis of variance (version 19) for comparison between the treatments according to Sokal and Rohlf [34]. Significant differences between the means of the parameters were investigated using Duncan’s multiple range tests, with a probability of ≤0.05. All of the previously mentioned measurable investigations were focused using SPSS software.

## 3. Results and Discussion

The chemical compositions of the *U. lactuca* and *U. fasciata* are shown in Table 1. The main component of *U. fasciata* and *U. lactuca* is the carbohydrate fraction, which occupies about 58.5 and 56.8 % on a dry-weight basis, followed by a protein which represents about 27.7 and 25.9 % of the dry weight. The lipid content didn’t exceed 1% in the two analyzed algal dry tissues, while the highest value of ash content was recorded for *U. lactuca*. Pauline et al. [8] and Taboada et al. [9] showed that *Ulva* sp. contains high quantities of beneficial-quality protein, carbohydrates, vitamins, and minerals. Research has displayed increased attention to the applications of polysaccharides due to their unique chemical and physical properties [35]. Ulvan is a form of sulphated heteropolysaccharide isolated from the cell walls of *Ulva* sp. marine green alga, the composition of which has been widely deliberated [36,37]. Ulvan is a sulphated heteropolysaccharide derived from the cell wall of *Ulva* sp. green seaweeds, the composition of which has been fiercely debated; it is a major biopolymeric fraction of the cell wall that is responsible for preserving the cell’s osmolar stability and defense [38]. *Ulva*. sp. has a high protein content, similar to legumes and grains, especially amaranth and soy [39]. The results revealed that *Ulva* sp has a low amount of lipids, and the ash content is much greater than that present in the higher plant; these results are in agreement with [40].

### 3.1. Physical Characterization of the Extracted Polysaccharides

#### Thermal Characteristics of the Extracted Polysaccharides

The thermal stability of the polysaccharide (Ulvan) extracted from the green algae *U. lactuca* and *U. fasciata* was investigated by thermogravimetric analysis (TGA), and is represented in Table 2. The first step of the thermal decomposition stage of the extracted polysaccharide from *U. lactuca* and *U. fasciata* occurred at around 92.55 and 112.46 °C, respectively. This decomposition is accompanied by about 10.794 and 12.935% mass loss, respectively. The second thermal decomposition took place at about 168.64 and 244.71 °C, respectively, with a loss of mass of about 4.975 and 14.053%, respectively. The third thermal decomposition took place at about 221.20 and 328.95 °C, respectively, with a loss of mass of about 13.471 and 10.251%, respectively. This decomposition continued with a further loss of mass of about 15.333 and 19.418% amid temperatures of about 665.93 and 677.72 °C, respectively. The remaining mass at the end of this process corresponded to the ash content in the sample [41]. According to the present thermogravimetric results, the extracted polysaccharides from the green algae *U. lactuca* and *U. fasciata* are thermally stable until about 169 and 245 °C, respectively. These values are in agreement with those recorded by Alves et al. [42].

### 3.2. Chemical Characterization of the Extracted Polysaccharide

**Figure 1:**
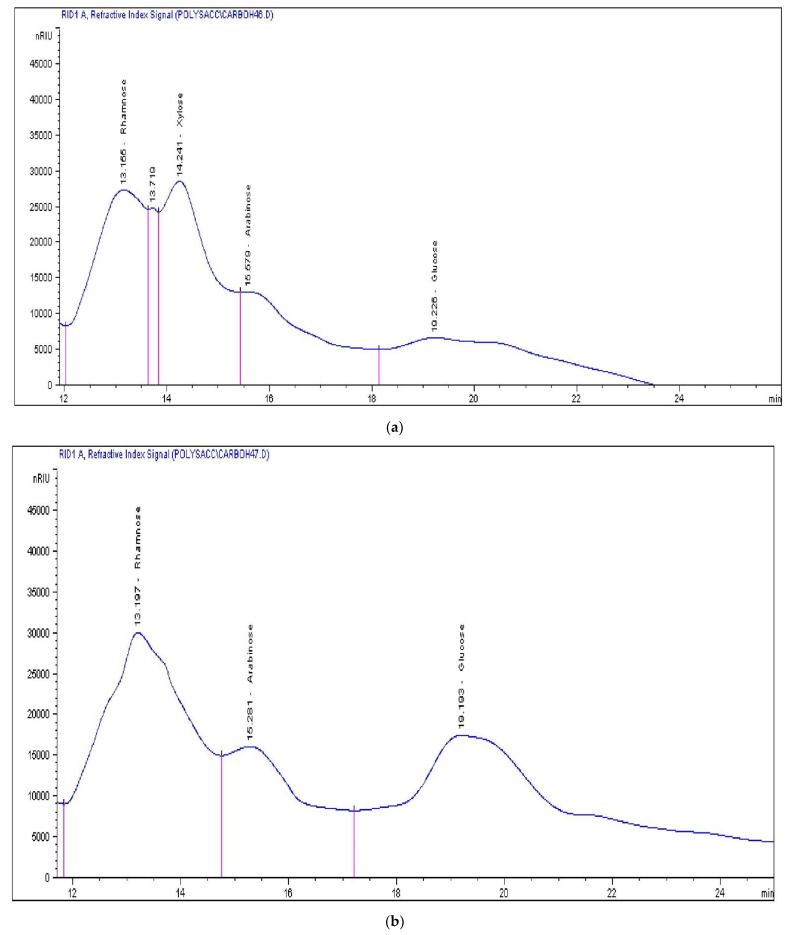
HPLC chromatograms of *Ulva lactuca* (**a**) (rhamnose, xylose, arabinose and glucose) and *Ulva fasciata* (**b**) (rhamnose, arabinose and glucose). X-axis: Retention times (min); Y-axis: Observed peak area (nRIU = the intensity of the refraction index signals).

The chemical compositions of the polysaccharides extracted from *U. lactuca* and *U. fasciata* is given in Figure 1 and Table 3. There were various types of sugars in the samples, such as xylose, glucose, rhamnose (in *U. lactuca* only), galactose, and arabinose. Each polysaccharide has a different composition than the others. Rhamnose, glucose, and galactose were the most abundant sugars. Ulvan is a form of sulphated heteropolysaccharide obtained from the cell walls of *Ulva* sp. green seaweeds, the composition of which has been extensively discussed [36,37]. Glucose is produced from hemicellulose and cellulose. The absence of fructose in the algal biomasses proved the absence of sucrose. The presence of rhamnose may be due to the existence of xylorhamnoglycuronane in this seaweed [36,37]. The variations in the sugar proportions cannot be attributed to the species or to the ecophysiological conditions [43].

### 3.3. Effect of Ulva’s Soluble Polysaccharide (Ulvan) on the Seed Germination and Seedling Growth of Zea Mays

#### 3.3.1. Germination Stage

**Figure 2:**
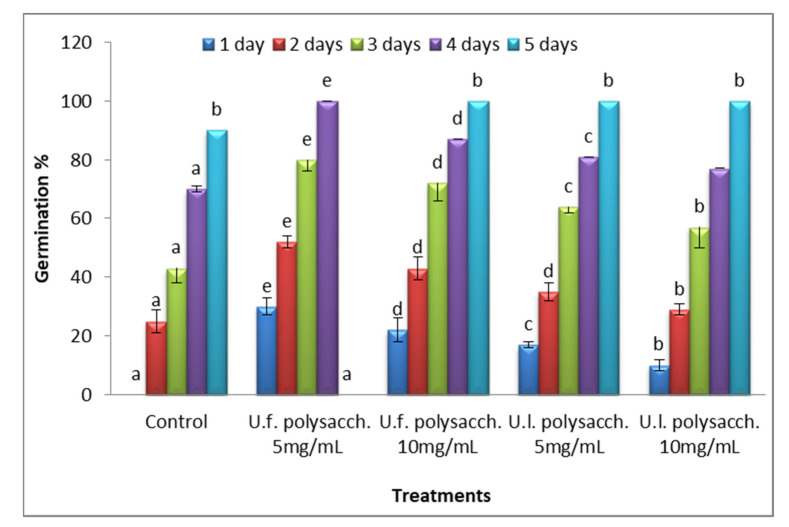
Effect of seed priming with different concentrations of soluble polysaccharides on the percentage of seed germination recorded daily during the germination period of *Zea mays*. The bars represent the standard error (different letters denote the significant value among the treatments on the same day (*p* value= 0.0000). U.f., *Ulva fasciata*; U.l., *Ulva lactuca*.

Investigating the biological activity, Figure 2 shows the changes in the germination percentage of the seeds treated with the extracted polysaccharides. The germination percentage increased progressively throughout the germination period (5 days) of all of the treatments. Compared to the control level, highly significant (*p* value = 0.000) increases in the germination percentage of *Zea mays* were found in the following descending order: *U. fasciata.* polysacch. 5 mg/mL *> U. fasciata.* polysacch. 10 mg/mL *> U. lactuca.* polysacch., 5 mg/mL *> U. lactuca.* polysacch., 10 mg/mL priming solution agent. Due to the high levels of organic matter, micro and macro elements, vitamins, and fatty acids, as well as being rich in growth regulators, liquid fertilizers originating from seaweeds are better than chemical fertilizers [44]. In the life cycle of flowering plants, seed germination is crucial. The germination process starts with the ingestion of dry seeds and ends with the embryo’s radical protrusion through the covering tissues [45]. Seaweed products have been shown to improve seed germination, improve nutrient absorption, enhance frost resistance, and help plants to resist phytopathological fungi and insect pests [46]. Furthermore, it was discovered that dilute extracts were more efficient than concentrated extracts [47]. Paulert et al. [48] found that using 10 mg/mL ulvan solute extracted from *Ulva fasciata* as a priming agent for *Phaseolus vulgaris* seeds increased the seed germination by about 24%. The present results follow those of Ramarajan et al. [49], who investigated the effect of seaweed extracts of *Ulva lactuca* and *Sargassum wightii* on bean seeds. They recorded that priming cluster bean seeds with 1% and 2% seaweed extracts enhanced the seed germination over the control level. The higher promotion effect was recorded at the lower concentration (1%) of seaweeds, and the ameliorating effect was attributed to *Ulva* rather than the *Sargassum* extract. This ameliorating effect may be attributed to the promotion effect of the water-soluble polysaccharide under investigation on a growth-promoting substance. The treated seeds with a green seaweed suspension had a significant stimulatory effect on germination, as they reduced the mean germination time at the optimum temperature in tomato and pepper seeds [50]. The active ingredients in seaweed extracts are efficient at low concentrations. Seaweed extracts enhance seed germination in several species, such as *Cajanus cajan* [51], *Trigonella foenum* [52], and *Capsicum annum* [53]. Seaweed extracts were found to improve seed germination in ornamental plants, tobacco, peas, and cotton at lower doses [54].

Seaweed liquid fertilizer (SLF) promoted the level of seed germination in green chilies and turnip, and it was found that minor concentrations of SLF promoted the germination percentage better than the superior concentration [13]. It was indicated that the promoting effect of seaweed extracts may originate from the higher level of seed moisture around the seeds [55]. In the present work, soluble polysaccharide priming agents (5 and 10 mg/mL of *U. fasciata* and *U. lactuca polysaccharide*) induced significant promotion effects on seed germination and seedling growth. It is noteworthy that these concentration doses lead to phytotoxicity. Cluz et al. [56] previously reported that the polysaccharide from *Ulva* spp. was non-phytotoxic.

#### 3.3.2. Radical Length

**Figure 3:**
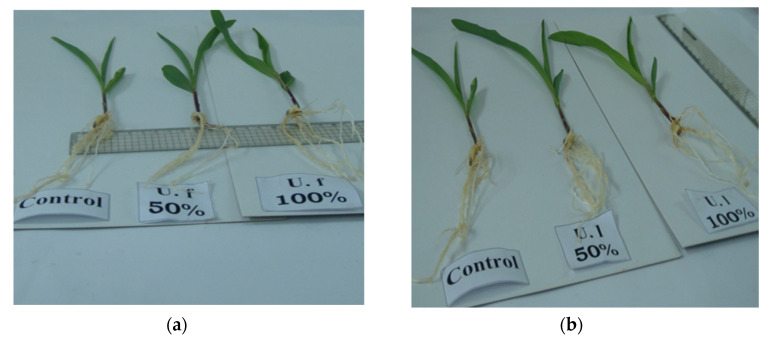
(**a**) Zea mays growing seedlings (U.f 50%: U.f. polysacch.5mg/mL, U.f 100%: U.f. polysacch. 10 mg/mL), (**b**) Zea mays growing seedlings (U.l 50%: U.l. polysacch. 5 mg/mL, U.l 100%: U.l. polysacch. 10 mg/mL).

**Figure 4:**
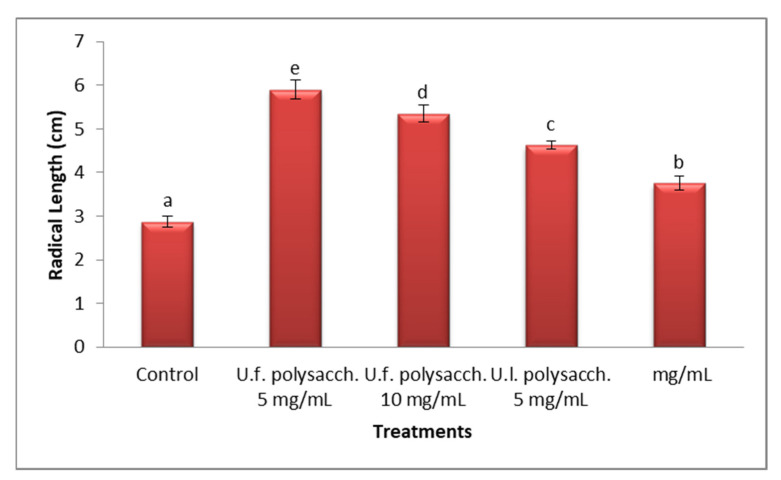
Effect of seed priming with different concentrations of soluble polysaccharides on the radical length of the seed germination of *Zea mays.* The bars represent the standard error. Different letters denote the significant values among the treatments (significant≤ 0.05). U.f., *Ulva fasciata;* U.l., *Ulva lactuca.*

Figure 3 and Figure 4 indicate the lengths of the radicles of the control and the treated seedlings of *Zea mays* appeared to increase progressively throughout the germination period (5 days). The *Zea mays* seedlings, primed with U.f. polysacch. 5 mg/mL and U.f. polysacch. 10 mg/mL, as compared with the control values, induced highly significant increases in radicle length, whereas significant increases were found upon priming with U.l. polysacch. 5 mg/mL and U.l. polysacch. 10 mg/mL. Kavipriy et al. [57] reported that seaweed extracts attained from seaweeds such as *Ulva lactuca*, *Caulerpa scalpelliformis, Sargassum plagiophyllum, Turbinaria conoides, Padina tetrastromatica,* and *Dictyota dichotama* strongly induce the seed germination and growth parameters of *Vigna radiata.* Among the four marine algae (*Ulva lactuca, Jania rubens, Laurencia obtusa* and *Sargassum vulgares*) which were tested, *Ulva lactuca* was the best alga for effectiveness in the promotion of banana plants’ growth [58].

#### 3.3.3. Morphological Criteria

**Figure 5:**
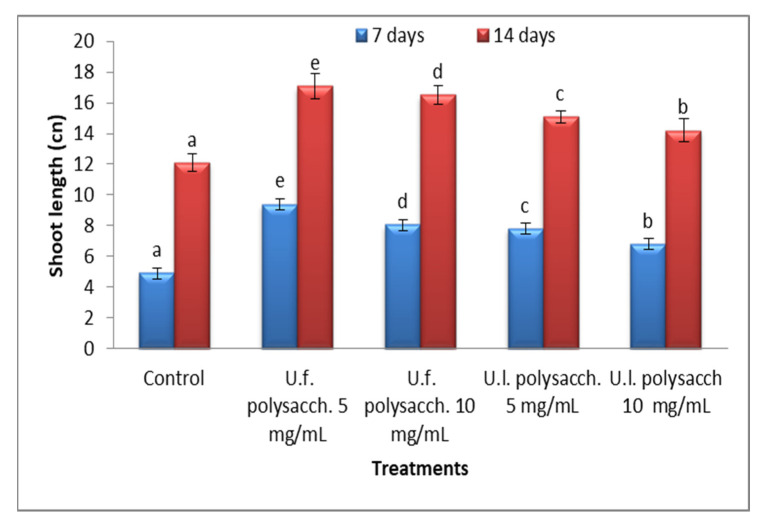
Effect of seed priming with different concentrations of soluble polysaccharide on the shoot height of *Zea mays,* measured at 7- and 14-day intervals during the germination period. The bars represent the standard error (different letters denote the significant values among the treatments on the same day). U.f., *Ulva fasciata;* U.l., *Ulva lactuca.*

**Figure 6:**
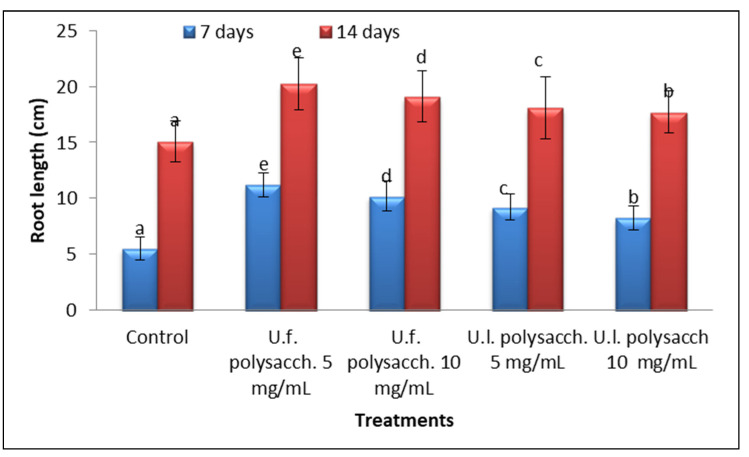
Effect of seed priming with different concentrations of soluble polysaccharides on the root length of *Zea mays* seedlings measured at 7- and 14-day intervals during the germination period. The bars represent the standard error (different letters denote the significant values among the treatments on the same day). U.f., *Ulva fasciata;* U.l., *Ulva lactuca.*

The data presented in Figure 5 and Figure 6 indicate the promoting action on the morphological criteria of the tested plants compared with the control values. The *Zea mays* seedlings primed with U.f. polysacch. 5 mg/mL and U.f. polysacch. 10 mg/mL, as compared with the control values, induced highly significant increases in shoot and root length, whereas a significant increase was found upon priming with both U.l. polysacch. 5 mg/mL and U.l. polysacch. 10 mg/mL.

#### 3.3.4. Fresh Weight

**Figure 7:**
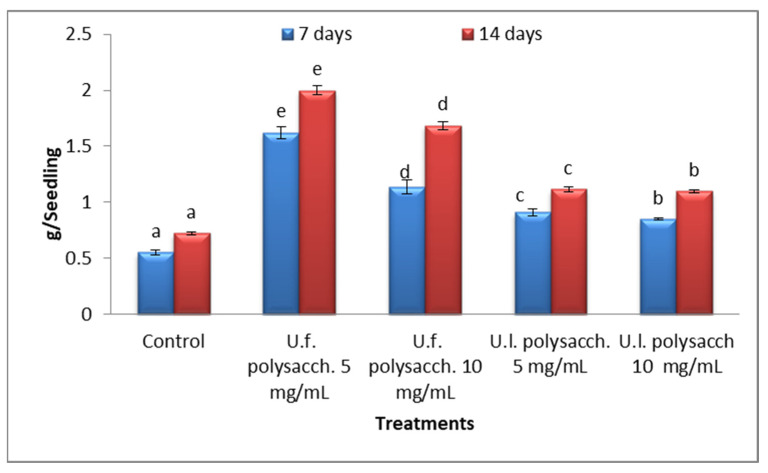
Effect of seed priming with different concentrations of soluble polysaccharides on the fresh weight of growing seedlings of *Zea mays* at 7- and 14-day intervals during the germination period. The bars represent the standard error (different letters denote the significant values among the treatments on the same day) U.f., *Ulva fasciata;* U.l., *Ulva lactuca.*

The data presented in Figure 7 shows that the fresh weights of the treated seedlings of *Zea mays* increased progressively throughout the experimental period (14 days). Highly significant increases in fresh weight were recorded in the following descending order: U.f. polysacch. 5 mg/mL *>* U.f. polysacch. 10 mg/mL > U.l. polysacch. 5 mg/mL > U.l. polysacch. 10 mg/mL.

#### 3.3.5. Dry Weight

**Figure 8:**
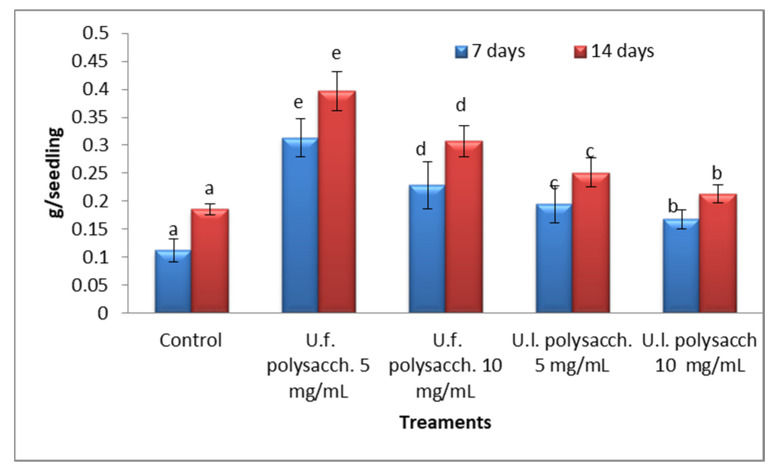
Effect of seed priming with different concentrations of soluble polysaccharides on the dry weight of growing seedlings of *Zea mays,* as determined at 7- and 14-day intervals during the germination period. The bars represent the standard error (different letters denote the significant value among the treatments on the same day). U.f., *Ulva fasciata;* U.l., *Ulva lactuca.*

Figure 8 shows the changes in the dry weight of the treated seeds of *Zea mays*. There appears to be a general progressive increase in the dry weight of the treated seeds of *Zea mays* throughout the germination period. All of the polysaccharide treatments induced significant increases in the dry weight of the treated seedlings, giving the following trend: U.f. polysacch. 5mg/mL > U.f. polysacch. 10mg/mL > U.l. polysacch. 5mg/mL > U.l. polysacch. 10mg/mL compared with the control values. Lower doses of seaweed liquid fertilizer (SLF) of *Chaetomorpha linum* (30%) were more effective regarding the growth parameters than higher doses (100%) when *Cajanus cajan* seeds were rinsed with it, along with lower concentrations of SLF extracted from *Stoechospermum marginatum* [59]. Similar effects were obtained with lower concentrations of seaweeds on the promotion of plants such as *Ascophyllum* and *Laminaria,* and accelerated maize growth [60]. *Padina* induced the maximum growth in *Cajanus cajan* [61], *Stoechosperum marginatum, Hypnea musciformis*, *Spatoglossum asperum*, and *Sargassum* with the growth of green chilies, pineapples, and turnips [62]. Selvam et al. [63] recorded that *Vigna mungo* seeds soaked with lower concentrations (1, 2.5, and 5 %) of seaweed extract prepared from the marine green algae *Ulva reticulate* showed higher rates of germination and enhanced shoot and root length, while the higher concentrations (7.5 and 10%) of the extract inhibited the germination.

#### 3.3.6. Changes in the Photosynthetic Pigments

**Figure 9:**
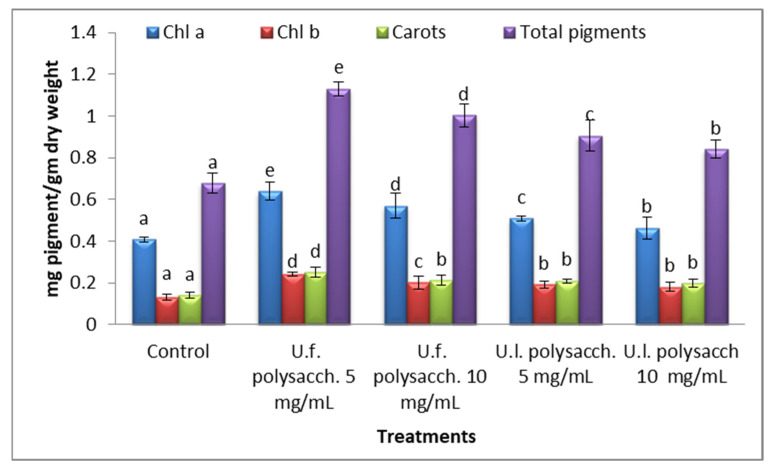
Effect of seed priming with different concentrations of soluble polysaccharides on the photosynthetic pigment contents of growing *Zea mays* seeds. The bars represent the standard error (different letters denote the significant values among treatments with the same pigments). U.f., *Ulva fasciata;* U.l., *Ulva lactuca.*

The results regarding the photosynthetic pigments are presented in Figure 9, and make it clear that the chlorophylls *a* and *b,* carotenoids, and total pigments at the vegetative stage of the treated *Zea mays* appeared to increase significantly in the following trend: U.f. polysacch., 5mg/mL, U.f., polysacch. 10 mg/mL, U.l. polysacch. 5 mg/mL, 10 mg/mL U.l., polysacch., respectively.

The increased level of chlorophyll observed in this study was supported by the findings of Blunden and Wildgoose [64], who reported a marked enhancement in the lateral root growth of potato plants as a result of the effect of seaweed extract. Stirk et al. [65] suggested that the secondary metabolites present in the polysaccharide extracts may stimulate growth by increasing the photosynthetic area of the treated seedlings.

Moreover, seaweed extracts such as brown algae have been added to many crops as promoters, with physiological promotions such as developed nutrient mobilization and partitioning, the advance of a vigorous root system, and improved chlorophyll content and leaf area. Christobel [66] deliberated the effects of seaweed liquid fertilizer (SLF) of *Sargassum wightii* on the germination, seedling growth, and phytochemical content of green gram (*Phaseolus aureus*). He found that a low level of seaweed liquid fertilizer of *Sargassum wightii* (1%) enhances the germination percentage and seedling growth, viz., root length, shoot length, leaves, chlorophyll content (chlorophyll a and b, and total chlorophyll), and enzyme activity. At higher levels (10 and 25%), the effect was inhibitive.

Thirumaran et al. [67] deliberated the influence of seaweed liquid fertilizer (SLF) on the growth and photosynthetic pigment of *Cyamopsis tetrogonolaba.* They found that the elevation of seed germination, growth and yield parameters, and photosynthetic pigment concentration were enhanced when treated with seaweed liquid fertilizer (SLF) at a 20% concentration, with or without chemical fertilizer. Francisca and Kalavathy [68] reported that aqueous extracts of *Sargassum wightii* and *Ulva lactuca* induced positive and significant responses in the growth and chlorophyll, protein, and carbohydrate contents of *Zea mays* plants. The increase in pigment production led to an improvement in the photosynthetic activity and carbohydrate content of the plant tissue. These findings could help to improve photosynthetic electron transport [69], pigment biosynthesis [70], and the interface with the thylakoid membrane surface [71]. Using seaweed extract as fertilizers increases the seed germination, seedling growth, and yield of the crop [72]. The micro-green alga *Scenedesmus obliquus* promoted banana plants’ growth with regard to shoot and root length and weight [73].

#### 3.3.7. Carbohydrate Contents

**Figure 10:**
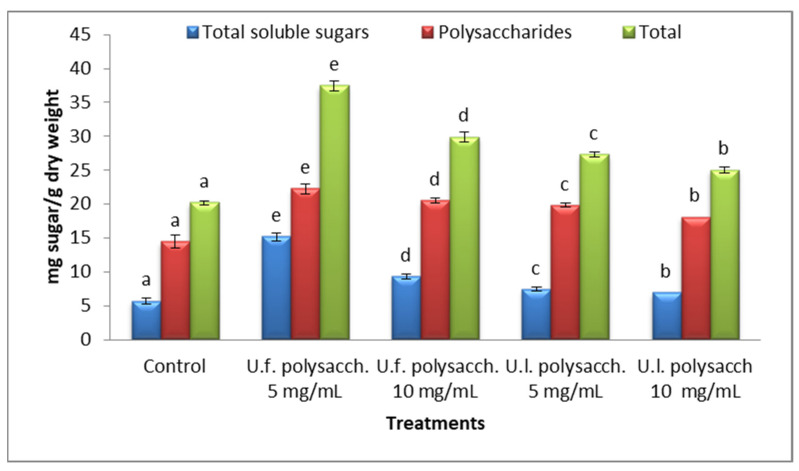
Effect of seed priming with different concentrations of soluble polysaccharides on the carbohydrate contents of growing *Zea mays* seeds. The bars represent the standard error (different letters denote the significant values among the treatments with the same parameter). U.f., *Ulva fasciata;* U.l., *Ulva lactuca*.

As compared with the control values, the perusal of the data presented in Figure 10 indicates that the highest productions of total carbohydrates and polysaccharides which were detected in the tested crop plant *Zea mays* with the treatments of U.f. polysacch. 5 mg/mL, U.f. polysacch. 10 mg/mL, and U.l. polysacch. 5 mg/mL, were 38.453, 29.9 and 27.27 mg sugar/gm dry weight, while the lowest production of carbohydrates which was recorded in the tested crop plant with the treatments of U.l. polysacch. 10 mg/mL was 25.26 mg sugar/gm dry weight. Our results coincide with those of El-Sheekh and El-Saied, [74], who reported that the total soluble sugars were elevated in both the shoot and root of *Vicia faba* with treatments of *Enteromorpha intestinalis, Cladophora dalmatica, Ulva lactuca, Corollina mediterranea, Pterocladia pinnata* and *Jania rubens* extracts. Abdel-Hamid et al. [75] reported that the consortium of *Chlorella vulgaris* and *Anabaena oryzae* enhanced Jerusalem artichokes’ growth, yield, and sugar contents.

#### 3.3.8. Changes in Enzymatic Activities

The full data of the changes in the enzymatic activities of the growing seedlings, as affected by different concentrations of priming soluble polysaccharides, are presented in Table 4. Compared with the control values, soluble polysaccharide treatments of U.f. polysacch. 5 mg/mL, U.f. polysacch. 10 mg/mL, U.l. polysacch. 5 mg/mL and U.l. polysacch. 10 mg/mL induced significant increases in the amylase activity of *Zea mays* in seedlings. The protease activity of germinating seedlings of *Zea mays* appeared to increase in response to all of the soluble polysaccharide treatments. The changes in the catalase and peroxidase contents of the investigated seedlings of *Zea mays* affected by priming with different concentrations of soluble polysaccharides were 38615.19 units/g tissue and 7.462 mg/g dry tissue, respectively. The catalase and peroxidase contents of 14-day-old treated seedlings of *Zea mays* exhibited significant escalations above the control level in response to soaking with soluble polysaccharides of *U. fasciata,* at 5 and 10 mg/mL, and *U.lactuca,* at 5 and 10 mg/mL, respectively. The amylase and protease activities of germinating *Zea mays* seeds showed a significant stimulatory effect in response to the priming agents of *Ulva* spp. soluble polysaccharide. Kato-Noguchi and Macias [76] found that the percentage of germination was correlated with the activity of α-amylase in lettuce seeds. The breakdown of proteins in the *Zea mays* germinating seeds is accomplished by the activities of protease and peptidase [77]. In germination, α-amylase is thought to play a key role in the conversion of reserve carbohydrates to soluble sugars [78]. Therefore, the stimulation of α-amylase is important in order to maintain an active respiratory metabolism, which permits the germination of plant seeds. The combinations of *Turbinaria ornata* and *Ulva reticulate* enhanced the germination and growth parameters of radish, green pea, and mung [79].

Table 5 shows the changes in the phenol content and total ascorbic acid content of the various investigated plant seedlings of *Zea mays,* as affected by priming in different concentrations of soluble polysaccharides. The total phenol content and total ascorbic acid content of 14-day-old treated *Zea mays* seedlings were significantly higher than the control level in response to priming with *U. fasciata* at 5 and 10 mg/mL, and *U.* *lactuca* at 5 mg/mL, respectively. The major contents of phenol and the total ascorbic acid were 4.835 mg/g dry weight and 1.658 µg/g dry weight with 5 mg/mL *U. fasciata,* respectively.

Catalase plays an important role in an antioxidant system with a specific function. Catalase degrades H_2_O_2_ (one of the very common ROS in plant tissues) into H_2_O and oxygen, where ASA serves as an electron donor to active H_2_O_2_ degradation by ascorbate peroxidase (APX) [80]. Catalase (CAT) activity was accompanied by improved germination performance in sunflower seeds [81], and also in purple and coneflower [82], Arabidopsis [83], and tomato [84]. Ulvan increased the peroxidase activity in beans (*Phaseolus vulgaris*) [85]. The treatment of higher plants with Ulvan prompts plant resistance and decreases the impact and severity of fungal infections [86]. Ulvan activity may be related to the presence of sulphate besides the presence of uronic acid and rhamnose in its contents, acting through the jasmonic acid pathway [87]. Several types of sulphated polysaccharides have been shown to exhibit antioxidant activity. Among them, Ulvan isolated from *Ulva pertusa* was found to play a vital role as a free radical scavenger in vitro, and exhibited antioxidant activity for the inhibition of oxidative damage in living organisms [88].

Phenols are very essential plant contents due to their scavenging capability and their hydroxyl groups [89]. A recent study demonstrated that a major positive relationship between the total phenol content and antioxidant activity was obtained in several higher plants [90]. The antioxidant and phenol contents increased in banana plants when they were treated with algal combinations of *Anabena oryza, Chlorella vulgaris* and *Scenedesmus obliquus* [91]. Ascorbic acid (vitamin C) is a water-soluble antioxidant molecule that acts as a basic substance in the cyclic pathway for the enzymatic detoxification of hydrogen peroxide; it acts directly to neutralize superoxide radicals, singlet oxygen, or superoxide [92,93]. The ascorbic acid (ASA) content of *Zea mays* seeds treated with the water-soluble polysaccharides of either *U. fasciata* or *U. lactuca* had significantly higher values than those of the control, as recorded in Table 5.

#### 3.3.9. Protein Content

**Figure 11:**
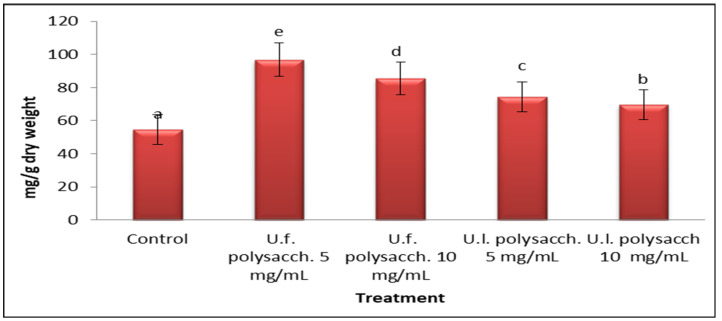
Effect of seed priming with different concentrations of soluble polysaccharides on the protein content of *Zea mays* growing seeds. The bars represent the standard error (the different letters denote the significant values among the treatments). U.f., *Ulva fasciata;* U.l., *Ulva lactuca.*

As the data presented in Figure 11 indicate, U.f. polysacch. 5 mg/mL, U.f. polysacch. 10 mg/mL, and U.l. polysacch. 5 mg/mL induced the highest production of protein content, and U.l. polysacch. 10 mg/mL induced the lowest of the different selected plant seedlings of *Zea mays;* the protein contents were 96.76, 85.49, 74.54 and 69.75, respectively. These results follow those of Christobel [66] and Asir and Saravanababu [94], who pointed out that there was an increase in the protein content at the lower concentrations of seaweed extract-treated plants. In the present study, the observed significant increases in the amylase and protease activities of both tested plant seedlings in response to the effect of the priming agents are in good accord with the rise in growth, as well as the carbohydrate and protein composition. The results show that the low concentrations of both U.f. polysacch and U.l. polysacch are more effective with regard to growth parameters, antioxdant activities and protein contents; this may be due to the high concentrations of polysaccharide having more viscosity than the low concentrations. These results are comparable to those obtained by Siddhanat et al. [95], who reported that increasing the concentration of Ulva polysaccharide solutions lead to an increase in viscosity. The lower viscosity of chitosan improved the stability of the soil aggregates by 100 to 200 times after just one cycle of wetting–drying [96].

#### 3.3.10. Protein Banding Pattern

##### Pattern of Total Soluble Proteins in *Zea mays* Samples

**Figure 12:**
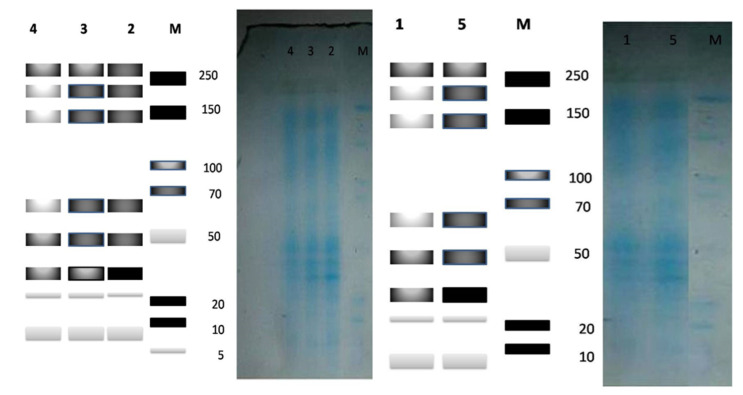
Photographs and zymograms of the protein patterns of *Zea mays.* M: Marker, 1: Control, 2: *U. fasciata* 5 mg/mL, 3: *U. fasciata* 10 mg/mL, 4: *U. lactuca* 5 mg/mL, 5: *U. lactuca* 10 mg/mL.

The changes in the protein banding pattern in response to the priming of *Zea* plants with polysaccharide solutions are shown in Figure 12. The scanning of the gel indicates the existence of forty bands with molecular weights (MW) ranging from 8.921 to 258.772 Kilo Dalton (KDa) for all of the investigated samples. All of the studied samples showed complete identity in the number of bands. All of the treatments were characterized by eight common bands at molecular weights of 8.921, 17.854, 22.537, 29.326, 29.326, 35.19, 138.807, 170.837, and 258.772 KDa, respectively. Polysaccharide supplementation increased the relative protein concentrations; this may be due to gibberellin-prompted processes during seed germination [97].

## 4. Conclusions

It can be concluded that the soluble extracted polysaccharides of *U. fasciata* and *U. lactuca* are sources of bioactive compounds with potential applications in agriculture as they enhance the antioxidant activity and carbohydrate and protein contents of *Zea mays. U. fasciata* contains the worst bioactive material, rather than *U. lactuca.* The lower concentrations of soluble polysaccharide are more effective with regard to the growth parameters, carbohydrates, protein contents and antioxidant activates of *Zea mays* than higher concentrations. Polysaccharide supplementation increased the relative protein concentrations of *Zea mays*. The application of seaweeds’ soluble polysaccharide extracts as biofertilizers is an eco-friendly approach for sustainable agriculture and the environment.

## Figures and Tables

**Table 1:** Chemical composition as a percentage of the dry weight of *Ulva lactuca* and *Ulva fasciata.*

Species	Carbohydrates	Protein	Lipids	Ash
*Ulva lactuca*	56.8 ± 4	25.9 ± 2.5	0.59 ± 0.02	16.3 ± 1.3
*Ulva fasciata*	58.5 ± 6	27.7 ± 3.3	0.70 ± 0.01	12.8 ± 2.2

**Table 2:** TGA analysis of the extracted polysaccharides from *U. fasciata* and *U. lactuca*.

*Ulva Lactuca*				*Ulva Fasciata*			
TS (°C)	TM (°C)	TE (°C)	Loss (%)	TS (°C)	TM(°C)	TE (°C)	Loss (%)
33.64	92.55	142.65	10.794	30.48	112.46	200.33	12.935
142.65	168.64	195.60	4.975	200.97	244.71	291.39	14.053
195.60	221.20	269.16	13.471	291.39	328.95	368.81	10.251
592.58	665.93	751.99	15.333	592.58	677.72	738.37	19.418

T_S_, T_M_ and T_E_: Start, middle and end temperatures, respectively. Loss (%): The percentage of weight loss.

**Table 3:** Monosaccharide composition (mg/g) of the polysaccharides extracted from the green seaweeds *Ulva lactuca* and *Ulva fasciata.*

Species	Xylose	Arabinose	Rhamnose	Glucose & Galactose
*Ulva lactuca*	62.3881	36.0013	90.1297	75.9949
*Ulva fasciata*	-	33.3524	146.9788	125.9704

**Table 4:** Effects of seed priming with different concentrations of soluble polysaccharides on the enzyme activities of growing seedlings of *Zea mays* seeds at the end of the germination period (5 days).

	Amylase	Protease	Catalase	Peroxidase
**Treatments**				
**Control**	100.18 ± 17 ^a^	60.93 ± 13 ^a^	18011.11 ± 300 ^a^	2.067 ± 0.12 ^a^
**U.f. polysacch. 5mg/mL**	447.34 ± 28 ^e^	482.81 ± 32 ^e^	38615.19 ± 57 ^e^	7.46 ± 0.21 ^e^
**U.f. polysacch. 10mg/mL**	370.81 ± 25 ^d^	447.18 ± 36 ^d^	34623.21 ± 55 ^d^	5.95 ± 0.19 ^d^
**U.l. polysacch. 5mg/mL**	308.57 ± 15 ^c^	315.93 ± 25 ^c^	29765.43 ± 53 ^c^	4.02 ± 0.09 ^c^
**U.l. polysacch. 10mg/mL**	223.26 ± 21 ^b^	248.43 ± 18 ^b^	25341.54 ± 51 ^b^	3.34 ± 0.16 ^b^
***p* value**	0.0011	0.0012	0.002	0.009

The amylase activity is calculated as the mg reducing sugars. min^−1^·g^−1^ fresh weight. The protease activity is calculated as the µg amino acids. min^−1^·g^−1^ fresh weight. The catalase content is given in units/g tissue, and the peroxidase content is given in mg/g dry tissue. Different letters denote the significant values among the treatments with each enzyme.

**Table 5:** Effect of seed priming with different concentrations of soluble polysaccharides on the ascorbic acid content (µg/g dry tissue) and total phenolic content (mg/g dry tissue) of *Zea mays*.

Treatment	Control	*U. fasciata*	*U. fasciata*	*U. lactuca*	*U.lactuca*	*p* Value
		5mg/mL	10mg/mL	5mg/mL	10mg/mL	
Ascorbic Acid	0.416 ± 0.022 ^a^	1.658 ± 0.054 ^e^	1.275 ± 0.062 ^d^	0.996 ± 0.033 ^c^	0.871 ± 0.012 ^b^	0.0000
Phenolic	1.443 ± 0.056 ^a^	4.835 ± 0.13 ^e^	4.016 ± 0.17 ^d^	3.38 ± 0.081 ^c^	2.892 ± 0.09 ^b^	0.0000

Different letters denote the significant values among the treatments.

## Data Availability

Not applicable.

## Sample Availability

Samples of the compounds are available by authors

## References

[1] Gibbon D., Pain A. (1985). Crops of Drier Regions of the Tropics.

[2] Subramaniyan V., Malliga. P. (2011). Effect of Cyanopith Biofertilizer as Basal and Spray on *Zea mays* (Corn). Int. J. Environ. Sci..

[3] Schwietzke S., Kim Y., Ximenes E., Mosier N., Ladisch M. (2009). Ethanol production from maize. Molecular Genetic Approaches to Maize Improvement.

[4] Mishra K.k., Vikram P., Yadaw R.B., Swamy M.B.P., Dixit S., Cruz M.T.S., Maturan P., Marker S., Kumar A. (2013). A locus with a consistent effect on grain yield under drought in rice. BMC Genet..

[5] Ahmed A.A.S. (2009). Cyanobacterial Application for the Improvement of Soil Fertility. Master Thesis.

[6] Chiellini E., Cinelli P., Ilieva V., Martera M. (2008). Biodegradable Termoplastic Composites Based on Polyvinyl Alcohol and Algae. Biomacromolecules.

[7] Chiellini E., Cinelli P., Ivanova I.V., Zimbardi F., Kanellopoulos N., de Wilde B., Pipino A., Anders B. (2009). Hybrid Composites Based on Fibres of Marine Origin. Int. J. Mater. Prod. Technol..

[8] Pauline S., Claire J., Elie D., Arsène I. (2006). Commercial applications of microalgae. J. Biosci. Bioeng..

[9] Taboada S., Garcia-Fernandez L.F., Bueno S., Vazquez J., Cuevas C., Avila. C. (2010). Antitumoral activity in Antarctic and Sub-Antarctic benthic organisms. Antarct. Sci..

[10] Divya K., Roja M.N., Padal S.B. (2015). Influence of seaweed liquid fertilizer of Ulva lactuca on the seed germination, growth, productivity of *Abelmoschus esculentus* (L). Int J Pharm. Res.

[11] Türkmen M., Su A. (2019). The effect of sea lettuce (*Ulva lactuca*) liquid fertilizer and zeolite combinations on the development of cucumber (*Cucumis sativus*). Turk. J. Agric. Food Sci. Technol..

[12] Safinaz A.F., Ragaa A.H. (2013). Effect of some red marine algae as biofertilizers on growth of maize (*Zea mays* L.) plants. Int. Food Res. J..

[13] Dhargalkar V.K., Untawale A.G. (1983). Some observations of the effect of SLF on higher plants. Indian J. Mar. Sci..

[14] Wajahatullah K.U., Rayirath P., Subramanian S., Jithesh M.N., Rayorath P., MarkHodges D., Critchley A.T., Craigie J.S., Norrie J., Prithiviraj B. (2009). Seaweed Extracts as Biostimulant of Plant Growth and Development. J. Plant. Growth. Regul..

[15] Norrie J., Keathley J.P. (2006). Benefit of Ascophyllum nodosum marine-plant extract ‘Thompson seedless’ grape production. (Proceeding softhe Xth International Symposiumon Plant Bioregulators in Fruit Production. Acta Hortic..

[16] Kloareg B., Quatrano R.S. (1988). Structure of the cell walls of marine algae and ecophysiological functions of the matrixpolysaccharides. Oceanogr. Mar. Biol. Annu. Rev..

[17] Dixon G.R., Walsh U.F. (2002). Suppressing *Pythium ultimum* induced damping-off in cabbage seedlings by biostimulation with proprietary liquid seaweed extracts managing soil-borne pathogens: A sound rhizosphere to improve productivity in intensive horticultural systems. Proceedings of the XXVIth Int. Hortic. Congr. Tor. Can..

[18] Ferreira L.G., Noseda M.D., Gonçalves A.G., Ducatti D.R.B., Fujii M.T., Duarte M.E.R. (2012). Chemical structure of the complex pyruvylated and sulfated agaran from the red seaweed *Palisada flagellifera* (Ceramiales, Rhodophyta). Carbohydr. Res..

[19] Wijesinghe W.A.J.P., Jeon Y.J. (2012). Enzyme-assistant extraction (EAE) of bioactive components: A useful approach for recovery of industrially important metabolites from seaweeds: A review. Fi-Toterapia.

[20] Vidanarachchi J.K., Iji P.A., Mikkelsen L.L., Sims I., Choct M. (2009). Isolation and characterization of water-soluble prebiotic compounds from Australian and New Zealand plants. Carbohydr. Polym..

[21] Ganapathy S.G., Sivakumar K. (2013). Effect of foliar spray from seaweed liquid fertilizer of *Ulva reticulata* (Forsk) on *Vigna mungo* L. and their elemental composition using SEM- energy dispersive spectroscopic analysis. Asian Pac. J. Reprod..

[22] Bourgougnon N., Lahaye M., Chermann J., Kornprobst J. (1993). Composition and antiviral activities of sulfated polysaccharide from *Schizymenia dubyi* (rodophyta, gigartinales). Bioorg. Med. Chem..

[23] Younesikelaki F.S., Ebrahimzadeh M.H., Desfardi M.K., Banala M., Marka R., Nanna R.S. (2016). Optimization of seed surface sterilization method and in vitro seed germination in *Althaea officinalis* (L.)-an important medicinal herb. Indian J. Sci. Technol..

[24] Dubois M., Gilles K.A., Hamilton J.K., Rebers P.A., Smith F. (1956). Colorimetric method for determination of sugars and related substances. Anal. Chem..

[25] Lowry O., Rosebrough A., Randell R. (1951). Protein measurement with Folin-Phenol reagent. J. Biol. Chem..

[26] Singleton V.L., Rossi J.A. (1965). Colorimetry of total phenolics with phosphomolybdic–phosphotungstic acid reagents. Am. J. Enol. Viticul..

[27] Stegman B.W., Shah A.A., Framchsen H., Elen K. (1988). Gel Electrophoresis and Isoelectric Focusing. Pantaphor Manual.

[28] Hemeida A. (1994). Cytological and Biochemical Genetic Studies in Fishes. Ph.D. Thesis.

[29] Chen G.-X., Asada K. (1989). Ascorbate peroxidase in tea leaves: Occurrence of two isozymes and the differences in their enzymatic and molecular properties. Plant Cell Physiol..

[30] Kang H.M., Saltveit M.E. (2001). Activity of enzymatic antioxidant defense systems in chilled and heat shocked cucumber seedling radicles. Physiol. Plant..

[31] Racusen D., Foote M. (1965). Protein synthesis in dark grown bean leaves. Can. J. Bot..

[32] Monroe J., Preiss J. (1990). Purification of amylase that accumulates in *Arabidopsis thaliana* mutants defective in starch metabolism. Plant Physiol..

[33] Anson M. (1938). Estimation of pepsin, trypsin, papain and cathepsin with hemoglobin. J. Gen. Physiol..

[34] Sokal R.R., Rohlf F.J. (1995). Biometry: The Principles and Practice of Statistics in Biological Research.

[35] Annette B., Jonas D., Henrik B., Lars N., Michael B., Stiig M., Birgit O., Carlos A., Peter D. (2011). Bioenergy potential of *Ulva lactuca*: Biomass yield, methane production and combustion. Bioresour. Technol..

[36] Lahaye M., Robic A. (2007). Structure and functional properties of ulvan, a polysaccharide from green seaweeds. Biomacromolecules.

[37] Robic A., Rondeau-Mouro C., Sassi J., Lerat Y., Lahaye M. (2009). Structure and interactions of ulvan in the cell wall of the marine green algae *Ulva rotundata* (Ulvales, Chlorophyceae). Carbohydr. Polym..

[38] Ortiz J., Romero N., Robert P., Araya J., Lopez Hernandez J., Bozzo C. (2006). Dietary fiber, amino acid, fatty acid and tocopherol contents of the edible seaweeds *Ulva lactuca* and *Durvillaea antarctica*. Food Chem..

[39] Norziah M., Ching Y. (2000). Nutritional composition of edible seaweed *Gracilaria changgi*. Food Chem..

[40] Ruperez P., Ahrazem O., Leal J. (2002). Potential antioxidant capacity of sulfated polysaccharides from the edibic marine brown seeweed *Fucus vesiculosus*. J. Agric. Food Chem..

[41] Anastasakis K., Ross A. (2011). Hydrothermal liquefaction of the brown macro-alga *Laminaria saccharina*: Effect of reaction conditions on product distribution and composition. Bioresour. Technol..

[42] Alves A., Caridade S., Mano J., Sousa R., Reis R. (2010). Extraction and physico-chemical characterization of a versatile biodegradable polysaccharide obtained from green algae. J. Carbohydr. Res..

[43] Lahaye M., Cimadevilla E., Kuhlenkamp R., Quemener B., Lognoné V., Dion P. (1999). Chemical composition and 13C NMR spectroscopic characterization of ulvans from *Ulva* (*Ulvales, Chlorophyta*). J. Appl. Phycol..

[44] Crouch I., Staden J. (1993). Effects of seaweed concentrate from *Ecklonia maxima* (Osbeck) paenfess on *Melodogyre incognita* infestation on tomato. J. Appl. Phycol..

[45] Foley E., Chao S., Horvath P., Dogramaci M., Anderson V. (2012). The transcriptomes of dormant leafy spurge seed under alternating temperature are differently affected by germination –enhancing pretreatment. J. Plant Physiol..

[46] Booth E. (1969). The manufacture and properties of liquid seaweed extracts. Proc. Int. Seaweed Symp..

[47] Bukhari S.S., Unttawale A.G. (1978). Seaweeds as liquid fertilizer and foliar spray. Seaweed Res. Utiln.

[48] Paulert R., Talamini V., Cassolato J., Duarte M., Noseda M., Smania A., Stadnik M. (2009). Effects of sulfated polysaccharide and alcoholic extracts from green seaweed *Ulva fasciata* on anthracnose severity and growth of common bean (*Phaseolus vulgaris* L.). J. Plant Dis. Prot..

[49] Ramarajan S., Raya S., Gandhi A. (2012). Effect of seaweed extracts mediated changes on the germination and pigment concentration of cluster bean (var. Pusa naubahar). J. Agric. Sci. Technol..

[50] Demir N., Dural B., Yildirim K. (2006). Effect of seaweed suspensions on seed germination of Tomoto, Pepper and Aubergine. J. Biol. Sci..

[51] Erulan V., Soundarapandian P., Thirumaran G., Ananthan G. (2009). Studies on the effect of *Sargassum polycystum* (C.Agardh, 1824) Extract on the growth and biochemical composition of *Cajanus cajan* (L.) Mill sp. American-Eurasian. J. Agric. Environ. Sci..

[52] Sabale A., Pise N. (2010). Effect of seaweed extracts (SWE) on germination of *Trigonella foenum*-graecum seeds. Bioinfolet.

[53] Sridhar S., Rengasamy R. (2012). The effect of seaweed liquid fertilizer of *Ulva lactuca* on *Capsicum annum*. Algol. Stud..

[54] Aitkin J., Senn J. (1965). Seaweed products as fertilizers and soil conditioners. Bot. Mar..

[55] Arumugam R., Anantharaman P. (2009). Effect of seaweed liquid fertilizer on growth and pigment concentration of *Abelmoschus esculentus* (l) medikus. J. Agron..

[56] Cluz S., Torregrossa C., Jacquet C., Lafitte J., Fournier J., Mercier L., Salamagne S., Briand X., Esquerre-Tuggaye M., Dumas B. (2004). Gene expression profiling and protection of *Medicago truncatula* against a fungal infection in response to an elicitor from green algae *Ulva* spp. Plant Cell Environ..

[57] Kavipriy R., Dhanalakshmi P., Jayashree N., Thangaraju N. (2011). Seaweed extract as a biostimulant for legume crop, green gram. J. Ecobiotechnol..

[58] El-Ansary M.S.M., Hamouda R.A. (2014). Biocontrol of root-knot nematode infected banana plants by some marine algae. Russ. J. Mar. Biol..

[59] Vijayanand V., Rathinavel S. (2004). Bio-fertilizing efficiency of seaweed liquid extract of *Hydroclathrus clathratus* on *Sorghum vulgare*. Seaweed Res. Util. Assoc..

[60] Stephenson W. (1974). Seaweeds in Agriculture and Horticulture. Reteaver, Peruma Valley.

[61] Mohan V.R., Venkataraman Kumar V., Murugeswari R., Muthuswami S. (1994). Effect of crude and commercial seaweed extracts on seed germination and seedling growth in *Cajanus cajan* L. Phykos.

[62] Dhargalkar V., Untawale A. (1980). Some observations of effect of seaweed liquid fertilizer on the higher plants. Proceedings of the National Workshop on Algal Systems.

[63] Selvam G., Balamurugan M., Thinakaran T., Sivakumar K. (2013). Developmental changes in the germination, growth and chlorophyllase activity of *Vigna mungo* L. using seaweed extract of *Ulva reticulata* forsskal. J. Pharm. Int. Res..

[64] Blunden G., Wildgoose P. (1977). The effect of aqueous seaweed extract and kinetin on potato yields. J. Sci. Food Agric..

[65] Stirk W., Arthur G., Lourens A., Novak O., Strnad M., Vanstaden J. (2004). Changes in cytokinin and auxin concentrations in seaweed concentrates when stored at an elevated temperature. J. Appl. Phycol..

[66] Christobel G. (2008). Effect of seaweed (*Sargassum wightii* L.) on the germination of green gram (*Phaseolus aureus* L.). J. Basic Appl. Biol..

[67] Thirumaran G., Arumugam M., Arumugam R., Anantharaman P. (2009). Effect of seaweed liquid fertilizer on growth and pigment concentration of *Cyamopsis tetrogonolaba* (L.) taub. Am. Eurasian J. Agron..

[68] Francisca P., Kalavathy S. (2011). A comparative study on the efficiency of two seaweed extracts as biostimulants on *Zea mays* L. J. Basic Appl. Biol..

[69] Bograh A., Gingras Y., Tajmir R., Carpentier R. (1997). The effects of spermine and spermidine on the structure of photosystem II proteins in relation to inhibition of electron transport. FEBS Lett..

[70] Younis M., El-Shahaby A., Abo-Hamed A., Haroun S. (1991). Plant growth, metabolism and adaptation in relation to stress conditions. XI. Modification of osmotic-stress-induced metabolic effects by GA3 or IAA in *Pisum sativum* plants. Acta Agron. Hung..

[71] Zheleva D., Tsonev T., Sergiev I., Karanov E. (1994). Protective effect of exogenous polyamines against atrazine in pea plants. J. Plant Growth Regul..

[72] Patel R.V., Pandya K.Y., Jasrai R.T., Brahmbhatt N. (2017). A review: Scope of utilizing seaweed as a biofertilizer in agriculture. Int. J. Adv. Res..

[73] El-Ansary M.S.M., Hamouda R.A., Ahmed-Farid O.A. (2021). Bioremediation of Oxamyl Compounds by Algae: Description and Traits of Root-Knot Nematode Control. Waste Biomass Valorization..

[74] El-Sheekh M., El-Saied A. (1999). Effect of seaweed extracts on seed germination, seedling growth and some metabolic processes of faba beans (*Vicia faba* L.). J. Phycol. Soc..

[75] Abdel-Hamid M.S., Hamouda R.A.E.F., Abd El-Aal H. (2022). Distinctive Application of the Consortium of *Chlorella vulgaris* and *Anabaena oryzae* toward different planting dates and climate change on Jerusalem Artichoke Yield. J. Plant. Growth. Regul..

[76] Kato-Noguchi H., Macias A. (2005). Effects of 6-methoxy-2-benzoxazolinone on the germination and a-amylase activity in lettuce seeds. J. Plant Physiol..

[77] Mikkonen A. (1986). Activities of some peptidases and proteinases in germinating kidney bean, *Phaseoulus vulgaris*. Physiol. Plant..

[78] Perata P., Guglielminetti L., Alpi A. (1997). Mobilization of endosperm reserves in cereal seeds under anoxia. Ann. Botony..

[79] Karthik T., Sarkar G., Babu S., Amalraj L.D., Jayasri M.A. (2020). Preparation and evaluation of liquid fertilizer from *Turbinaria ornata* and *Ulva Reticul*. Biocatal. Agric. Biotechnol..

[80] Hassanein A., Bassuony F., Baraka D., Khalil R. (2009). Physiological effects of nicotinamide and ascrobic acid on *Zea mays* plant grown under salinity stress. I-Changes in growth, some relevant metabolic activities and oxidative defense systems. J. Agric. Biol. Sci..

[81] Bailly C., Bogatek-Leszczynska R., Come D., Corbineau F. (2002). Changes in activities of antioxidant enzymes and lipoxygenase during growth of sunflower seedlings from seeds of different vigour. Seed Sci. Res..

[82] Dohlert D., Stanley H., Duke A., Anderson L. (1982). Beta-amylases from alfalfa roots. J. Plant. Physiol..

[83] Chaparzadeh N., Amico D., Khavari- Najad A., Izzo R., Navarizzo F. (2004). Antioxidative responses of *Calendula officinalis* under salinity conditions. J. Plant Physiol. Biochem..

[84] El-Bassiony H. (2005). Physiological responses of wheat to salinity alleviation by nicotinamide and tryptophan. Int. J. Agric. Biol..

[85] Freitas M., Stadnik M. (2012). Race-specific and ulvan-induced defense responses in bean (*Phaseolus vulgaris*) aginst *Colletrichum lindemuthianum*. Physiol. Mol. Plant. Pathol..

[86] Araújo L., Stadnik M., Borsato L., Valdebenito-Sanhueza R. (2008). Potassium phosphite and ulvan in the control of ‘Gala’ leaf spot on apple. Trop. Plant. Pathol..

[87] Montealegre J., López C., Stadnik M., Henríquez J., Herrera R., Polanco R., Piero R., Pérez L. (2010). Control of grey rot of apple fruits by biologically active natural products. Trop. Plant. Pathol..

[88] Jaulneau V., Lafitte C., Jacquet C., Fournier S., Salamagne S., Briand X., Esquerré-Tugayé M., Dumas B. (2010). Ulvan, a sulfated polysaccharide from green algae, activates plant immunity through the jasmonic acid signaling pathway. J. Biomed. Biotechnol..

[89] Qi H., Zhang Q., Zhao T., Chen R., Zhang H., Niu X., Li Z. (2005). Antioxidant activity of different sulfate content derivatives of polysaccharide extracted from *Ulva pertusa* (Chlorophyta) in vitro. Int. J. Biol. Macromol..

[90] Yu L., Haley S., Perret J., Harris M., Wilson J., Aian M. (2002). Free radical scavenging properties of wheat extracts. J. Agric. Food Chem..

[91] Nurhanan A., Rosli W., Mohsin S. (2012). Total polyphenol content and free radical scavenging activity of cornsilk (*Zea mays* hairs). Sains Malays..

[92] Hamouda R.A., El-Ansary M.S.M. (2017). Potential of Plant-Parasitic Nematode Control in Banana Plants by Microalgae as a New Approach Towards Resistance. Egypt. J. Biol. Pest Control..

[93] Noctor G., Foyer H. (1998). Ascorbate and glutathione: Keeping active oxygen under Control. Annu. Revission Plant Physiol. Plant Mol. Biol..

[94] Asir S., Saravanababu S. (2004). Studies on the effect of seaweed extract in *Oryza sativa* var. Ambai-16 during senescence. Seaweed Res. Util. Assoc..

[95] Siddhanat A., Goswami A., Ramavat B., Mody K., Mairh O. (2001). Water soluble polysaccharides of marine algal species of Ulva (Ulvales, Chlorophyta) of Indian waters. Indian J. Mar. Sci..

[96] Adamczuk A., Kercheva M., Hristova M., Jozefaciuk G. (2021). Impact of Chitosan on Water Stability and Wettability of Soils. Materials.

[97] Jones H., Davies W., Davies W., Jones H. (1991). A Perspective on ABA research in the 1990s. Abscisic Acid Physiology and Biochemistry.

